# Enceladus Plume Structure and Time Variability: Comparison of Cassini Observations

**DOI:** 10.1089/ast.2017.1647

**Published:** 2017-09-01

**Authors:** Ben D. Teolis, Mark E. Perry, Candice J. Hansen, J. Hunter Waite, Carolyn C. Porco, John R. Spencer, Carly J. A. Howett

**Affiliations:** ^1^ Space Science Division, Southwest Research Institute, San Antonio, Texas.; ^2^ Johns Hopkins University, Applied Physics Laboratory, Laurel, Maryland.; ^3^ Planetary Science Institute, Tucson, Arizona.; ^4^ University of California, Berkeley, California.; ^5^ CICLOPS, Space Science Institute, Boulder, Colorado.; ^6^ Southwest Research Institute, Boulder, Colorado.

## Abstract

During three low-altitude (99, 66, 66 km) flybys through the Enceladus plume in 2010 and 2011, Cassini's ion neutral mass spectrometer (INMS) made its first high spatial resolution measurements of the plume's gas density and distribution, detecting *in situ* the individual gas jets within the broad plume. Since those flybys, more detailed Imaging Science Subsystem (ISS) imaging observations of the plume's icy component have been reported, which constrain the locations and orientations of the numerous gas/grain jets. In the present study, we used these ISS imaging results, together with ultraviolet imaging spectrograph stellar and solar occultation measurements and modeling of the three-dimensional structure of the vapor cloud, to constrain the magnitudes, velocities, and time variability of the plume gas sources from the INMS data. Our results confirm a mixture of both low and high Mach gas emission from Enceladus' surface tiger stripes, with gas accelerated as fast as Mach 10 before escaping the surface. The vapor source fluxes and jet intensities/densities vary dramatically and stochastically, up to a factor 10, both spatially along the tiger stripes and over time between flyby observations. This complex spatial variability and dynamics may result from time-variable tidal stress fields interacting with subsurface fissure geometry and tortuosity beyond detectability, including changing gas pathways to the surface, and fluid flow and boiling in response evolving lithostatic stress conditions. The total plume gas source has 30% uncertainty depending on the contributions assumed for adiabatic and nonadiabatic gas expansion/acceleration to the high Mach emission. The overall vapor plume source rate exhibits stochastic time variability up to a factor ∼5 between observations, reflecting that found in the individual gas sources/jets. Key Words: Cassini at Saturn—Geysers—Enceladus—Gas dynamics—Icy satellites. Astrobiology 17, 926–940.

## 1. Introduction

The Cassini spacecraft's 2005 discovery of geyser emissions at Enceladus' south polar region (Dougherty *et al.*, 2006; Hansen *et al.*, 2006; Spahn *et al.*, 2006; Tokar *et al.*, 2006; Waite *et al.*, 2006), from surface hot spots along parallel, elongated “tiger stripe” surface troughs (Porco *et al.*, 2006; Spencer *et al.*, 2006) was a major milestone in understanding both the physics of Saturn system and the likelihood and frequency of present-day geological activity at solar system icy satellites. The plumes may be fed by fracturing, exposure, and degassing of crustal clathrate hydrate (Kieffer *et al.*, 2006) and/or vaporization of liquid water (Schmidt *et al.*, 2008) beneath the icy crust (Iess *et al.*, 2014; Thomas *et al.*, 2016), with vapor and grains (Postberg *et al.*, 2009) possibly propelled to the surface and cooled by boiling (Porco *et al.*, 2006; Brilliantov *et al.*, 2008; Kite and Rubin, 2016; Nakajima and Ingersoll, 2016) and pressure-driven gas expansion (Matson *et al.*, 2012; Yeoh *et al.*, 2015) through crustal fissures. Cassini observations over the last decade have since yielded details about the plume structure, composition, and variability, with analysis of Cassini imaging (Porco *et al.*, 2014), together with stellar and solar occultation data (Hansen *et al.*, 2008, 2011, 2017) that suggest the presence of multiple high-speed narrow jets of water vapor, ice, and salt-bearing grains imbedded within a broad plume that extends thousands of kilometers from Enceladus. The most robust jets form the faint extended arcuate tendrils seen in Cassini high solar phase images in the vicinity of Enceladus that eventually form the E ring (Mitchell *et al.*, 2015). Observations of individual jets changing—turning “on” and “off”—between different imaging observations (Porco *et al.*, 2014) and periodic variability in the total plume correlated to Enceladus' orbital position (Hedman *et al.*, 2013) indicate a dynamic plume modulated by tidally driven compression and expansion of the surface fissures (Hurford *et al.*, 2007; Nimmo *et al.*, 2014). Cassini's cosmic dust analyzer (Spahn *et al.*, 2006), together with multiple other instruments (Jones *et al.*, 2009; Yaroshenko *et al.*, 2009; Teolis *et al.*, 2010), have revealed the presence of both water ice-rich and salt-rich grains with a broad range of sizes and compositions (Postberg *et al.*, 2011) that range from charged nanometer-sized grains (Dong *et al.*, 2015), which are picked up by Saturn's electric and magnetic fields (Meier *et al.*, 2014; Mitchell *et al.*, 2015), escape (along with the gas) Enceladus' gravity, and supply material to Saturn's E-ring (Porco *et al.*, 2006; Kempf *et al.*, 2008), to micron-sized grains that fall back to the surface (Porco *et al.*, 2006; Hedman *et al.*, 2009; Kempf *et al.*, 2010). Cassini ion neutral mass spectrometer (INMS) measurements of the plume vapor, acquired *in situ* during multiple flybys directly through the plume (Teolis *et al.*, 2010), have shown the presence (in addition to H_2_O as the primary constituent) of CO_2_; possible CH_4_, NH_3_, and H_2_ (Bouquet *et al.*, 2015; Waite *et al.*, 2017); and large organic molecules (Waite *et al.*, 2009, 2011), giving a glimpse of the complex subsurface oceanic composition and chemistry. During several recent low-altitude (<100 km) plume traversals (Table 1), INMS also measured the plume vapor distribution encountered along the flyby trajectories (Perry *et al.*, 2015), including observations of the broad vapor cloud and discrete gas sources.

**Table 1.** Summary of Ion Neutral Mass Spectrometer and Ultraviolet Imaging Spectrograph Enceladus Flybys/Observations

*Date*	*Time*	*Flyby*	*Altitude*	*Speed*	*Description*
October 24, 2007	17:07:21 UTC		636442 km	22.6 km/s	Zeta Orionis, stellar UVIS, horizontal across plume, 15 km ray height
March 12, 2008	19:06:12	E3	50	14.4	Steeply inclined, fast pass north–south, outbound along the plume
October 9, 2008	19:06:40	E5	28	17.7	Steeply inclined, fast pass north–south, outbound along the plume
November 2, 2009	07:41:58	E7	91	7.74	Horizontal, slow pass, perpendicular to stripes, INMS low res
May 18, 2010	06:01:17	E10	429	6.55	Solar UVIS, horizontal across plume, 14 km min ray height
October 1, 2011	13:52:26	E14	90	7.43	Horizontal, slow pass, parallel to stripes, INMS high res
March 27, 2012	18:30:09	E17	66	7.5	Horizontal, slow pass, parallel to stripes, INMS high res
April 14, 2012	14:01:38	E18	66	7.5	Horizontal, slow pass, parallel to stripes, INMS high res

Times and altitudes are Cassini closest approach to Enceladus (INMS), or closest approach of the line-of-sight to the Enceladus limb (UVIS). This work uses data from low-altitude, high-resolution E14–18 flybys, the low resolution E7 data, and the UVIS zeta Orionis and Solar occultations.

INMS, ion neutral mass spectrometer; UVIS, ultraviolet imaging spectrograph.

In the present study, we used these INMS plume structure measurements, together with ultraviolet imaging spectrograph (UVIS) stellar and solar plume occultations and imaging of the grain jets, to constrain the properties (locations, magnitudes, and gas velocity) and time variability of the plume surface sources. Initial observations of the gas jets by Cassini's UVIS during a plume occultation of the star zeta Orionis on October 24, 2007 (Hansen *et al.*, 2008) showed fine structure in the water vapor density on the scale of a few kilometers in the plume, suggesting the presence of supersonic gas jets with thermal Mach numbers (the ratio of gas bulk and thermal velocities) of 1.5 ± 0.2. UVIS has since carried out several additional stellar occultations and one solar occultation during the May 18, 2010 E10 flyby with exceptionally good signal-to-noise (Hansen *et al.*, 2011) ratio, enabling multiple narrow, high Mach number jets to be discerned. However, the INMS detection of the individual gas jets required several attempts over multiple flybys, during which a number of measurement and instrumental challenges had to be overcome.

The E3 and E5 flybys on March 12, 2008 and October 9, 2008 were the first plume traversals for which INMS was aimed toward the spacecraft direction of motion to sample and measure the gas density and composition, as shown in Table 1. Both flybys took place along similar north to south trajectories and thereby encountered the plume after closest approach, sampling the plume density and composition as the spacecraft traveled outbound from the south polar region. In their analysis of these two flybys, Teolis *et al.* (2010) modeled water vapor adsorption on the walls of the INMS gas inlet thermalization antechamber and found that such sticking introduced a time delay and distortion in the INMS H_2_O data. They determined that “nonsticky” plume species, including CO_2_ vapor, yielded a signal in INMS more representative of the plume density versus position along Cassini's trajectory. The CO_2_ E3 and E5 data show an approximate inverse square decay of the plume density with distance from the south polar terrain, which is consistent with collisionless vapor expansion from Enceladus well in excess of the 240 m/s escape speed. Following up on early UVIS-based modeling, Burger *et al.* (2007), Tian *et al.* (2007), Tenishev *et al.* (2010), Dong *et al.* (2011), Tenishev *et al.*, (2014), Hurley *et al.* (2015), and Yeoh *et al.* (2017) applied analytical and Monte Carlo modeling to estimate plume source properties on the basis of these INMS data, that is, source rate and gas velocity, by fitting to these data the eight major grain jets identified from preliminary, low-resolution Cassini imaging (Spitale and Porco, 2007). Smith *et al.* (2010) combined Monte Carlo models of the plume and Saturnian magnetosphere and concluded (similarly with Dong *et al.* [2011], Tenishev *et al.* [2014], and Yeoh *et al.* [2017]) that the INMS data were consistent with an increase, by a factor ∼4, in the plume source rate from the E3 to the E5 flyby. Tenishev *et al.* (2014) also incorporated INMS E7 and UVIS stellar and solar occultation data into their modeling, and found that additional gas sources distributed along the tiger stripes were necessary to explain the observed gas distribution.

**FIG. 1.**
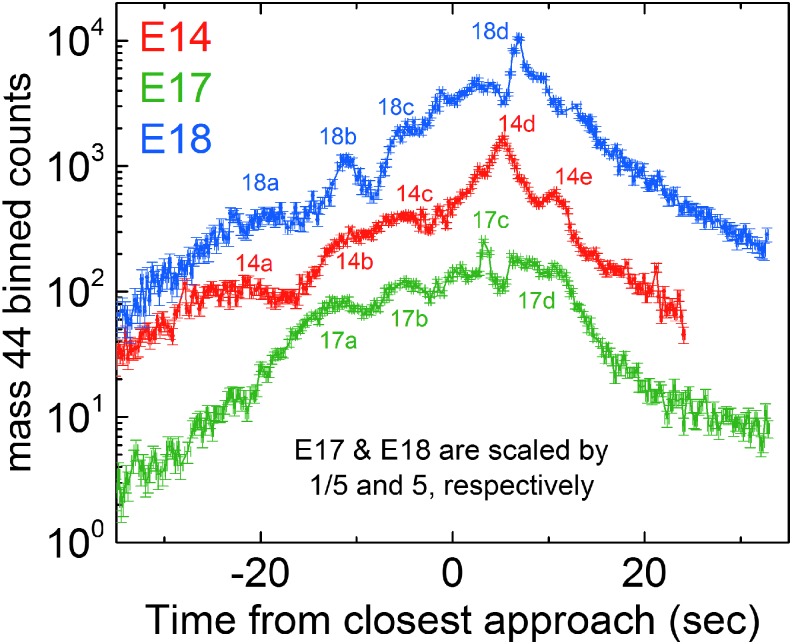
E14, E17, and E18 flyby data from the INMS mass 44 (CO_2_) channel (Perry *et al.*, 2015) showing counts versus time from point of closest approach (approximately over the Enceladus south pole), showing (i) a broad region of high density corresponding to the diffuse plume, and (ii) individual peaks (several of which have been labeled) due to plume structure. Data too long after CA are not shown since postencounter residual gas in the instrument starts to dominate the signal. INMS, ion neutral mass spectrometer. Color images available online at www.liebertonline.com/ast

**FIG. 2.**
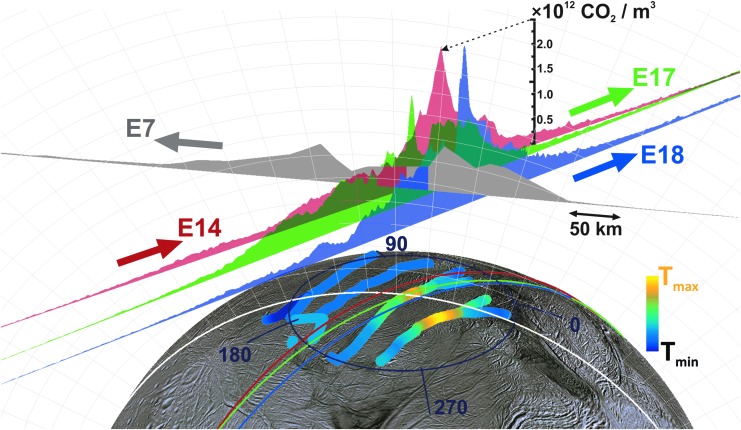
To scale 3D representation of the E14, 17, 18, and also (lower resolution) E7 INMS mass channel 44 data (Perry *et al.*, 2015), with vertical areas representing (in linear scale) the CO_2_ density, and the flat base of the areas corresponding to the Cassini trajectories. Lines across the surface are the ground tracks. The tiger stripes are colored according to the relative temperature estimated by CIRS (Spencer *et al.*, 2013). 3D, three-dimensional. Color images available online at www.liebertonline.com/ast

Beginning with the 91 km E7 flyby on November 2, 2009, the Cassini spacecraft carried out a series of low-altitude (<100 km) traversals over the south polar terrain, directly through the plume and sufficiently close to the tiger stripes to sample the detailed spatial distribution/structure of vapor in the gas jets. During these flybys, only the most abundant nonsticky plume species CO_2_ (with an ∼0.5% mixing ratio [Bouquet *et al.*, 2015]) had sufficient signal to noise in INMS data to enable detection of local density variations due to jets along Cassini's trajectory. At E7, INMS was programmed (following common practice) to sample all molecular masses with roughly equal cadence, ∼1.5 s, an approach intended to provide simultaneous density and compositional information along Cassini's trajectory. Unfortunately, 1.5 s time resolution translated (for the 7.7 km/s flyby speed) to a 12 km spatial resolution between CO_2_ measurements, which provided only poor resolution of the jets in the E7 data (Perry *et al.*, 2015). The INMS team, therefore, adjusted the measurement strategy on the later E14, E17, and E18 flybys, concentrating the INMS mass scans on the 44 amu CO_2_ channels, which yielded CO_2_ density data at a much higher 0.25 s temporal (1.9 km spatial) resolution. As shown in Figure 1, CO_2_ data from E14, E17, and E18 clearly resolved density variations indicative of plume structure, such as gas jets, along Cassini's trajectories. In Figure 2, we show a three-dimensional (3D) projection of these data over the Enceladus south polar terrain to illustrate how the jet structure observed by INMS is spatially distributed relative to the tiger stripes. Using these E14, E17, and E18 INMS data, Hurley *et al.* (2015) compared Monte Carlo plume models fed by (1) the eight (Spitale and Porco, 2007) sources and (2) a source continuously distributed along the tiger stripes, and concluded that data are best explained by a continuous source with location-dependent emission strength. In this study, we test the viability of a plume model fed by the 98 jets identified and precisely located in high-resolution imaging by Porco *et al.* (2014), with spatially and temporally variable vapor jet sources consisting of both a broad (slow, isotropic emission) and a sharp (fast, directed emission) component.

## 2. Analysis

Constraining the complete 3D plume structure solely on the basis of only a few INMS flybys is challenging, as multiple combinations of jet pointing directions and intensities can fit the data, and the jets may also be time variable. Time changing tidal stress fields in Enceladus' crust that act to open and close the surface fissures (Nimmo *et al.*, 2014) and that are thought to drive the plume's orbital position-dependent time variability (Hedman *et al.*, 2013) may also cause temporal changes in jet intensities unique to the individual jets, depending on local details of the subsurface fissure geometry beyond detectability. For many years, understanding of the jet 3D structure was limited to the preliminary imaging-based analysis of the grain jets by Spitale and Porco (2007) suggesting eight primary jet sources. Sufficient and high-resolution imaging data was eventually acquired to enable a more detailed analysis of the plume grain distribution, which suggested the presence of more than 98 individual jets, with a possible contribution of quasi-uniformly distributed interjet emission along the tiger stripes (Porco *et al.*, 2014). Using an alternate model, Spitale *et al.* (2015) hypothesized that the eruptions predominately take the form of continuous curtains of material, with very few collimated jets. This alternative has since been refuted (Porco *et al.*, 2015), but we address both models here. The grains are accelerated and carried in the vapor flow and, therefore, the locations and pointing directions (if not the spreading) of the vapor and imaged grain jets should coincide within tens of kilometers to the surface sources (the micron-sized grains decouple from the gas flow within 10 vent diameters, that is, within a few meters [Yeoh *et al.*, 2015]). Accordingly, the locations and directions as determined by Porco *et al.* (2014) can, in principle, provide a major constraint on UVIS- and INMS-based plume modeling. Using the UVIS data, Portyankina *et al.* (2016) began modeling the plume with all 98 (Porco *et al.*, 2014) jets, adopting a Monte Carlo modeling approach. Here, we combine UVIS with INMS observations to constrain the vapor emission intensity and velocity, assuming as sources, where applicable, the 98 particle jets, using an analytical modeling approach computationally efficient for analyzing such a large number of plume sources.

We approximate the gas velocity distribution as Maxwellian plus a source bulk velocity, which is computationally expedient since the gas density versus position *n* (*r*, θ, *S*, *M*, *v_M_*) of such a drifted Maxwellian distribution (with *r*, θ, *S*, *M*, *v_M_* the distance to source, angle from the jet axis, source flux, thermal Mach number, and gas thermal speed at the vent exit, respectively) is readily calculated with an analytical expression as applied by Dong *et al.* (2011) and Tenishev *et al.* (2014) (see Dong *et al.* [2011] for a full derivation):

\begin{align*}
{ n_ { M , i } } = & { \Omega _i } \frac { 1 }  { { 2 \pi { r^2 }
R } } \left\{ { \left( { 2 { \pi ^ { - 1 / 2 } } M \; \cos \;
\theta } \right) { e^ { - { M^2 } } } } \right.\\ & \left. +
\left( { 1 + 2 { M^2 } \; { { \cos } ^2 } \; \theta } \right)
\left( { 1 + erf ( M \; \cos \; \theta ) } \right) { e^ { - { M^2
} { { \sin } ^2 } \theta } }  \right\} \tag {1{\rm a}}
\end{align*}

\begin{align*}
R =  \left( {2{ \pi ^{ - 1 / 2}} - {M^{ - 1}}} \right) {e^{ -
{M^2}}} + \left( {2M + {M^{ - 1}}} \right) \left[ {1 + erf ( M ) }
\right] \tag{1{\rm b}}
\end{align*}

\begin{align*}
{ \Omega _i} = { \sigma _i} / {v_M} \; ( { \rm{model}} \;{
\rm{fitting}} \;{ \rm{parameter}} ) \tag{1{\rm c}}
\end{align*}

**FIG. 3.**
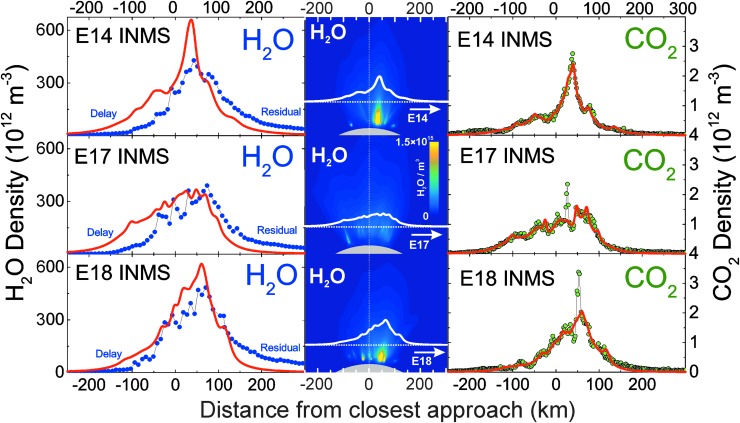
Right: model fits (red lines) to the INMS-measured CO_2_ densities (green points) versus distance along Cassini's trajectory, with the (Porco *et al.*, 2014) jets as the constraint. Left: INMS-measured H_2_O densities (blue points), and the CO_2_-based model prediction for the H_2_O density (red line). The prediction takes into account the relative mixing ratios of CO_2_ (∼0.24%, 0.37%, and 0.24% at E14, 17, and 18) and H_2_O (∼90%) in the plume, and corrects for the anticipated (m_CO2_/m_H2O_)^1/2^ thermal Mach number mass dependence (Hurley *et al.*, 2015; Perry *et al.*, 2015). Measured and modeled H_2_O disagree due to H_2_O adsorption in the INMS, which distorts the signal by (1) delaying H_2_O transmission through the gas inlet to the ion source and (2) causing residual H_2_O to persist in the instrument even after Cassini has exited the plume. Center: model plume density cross sections for each flyby in the plane subtended by Cassini's trajectory (dotted line) about Enceladus' center, with the solid white lines (same as red lines on left) showing the model density profile versus position. Note that densities decrease near the surface since the plane shown cuts between tiger stripes and jets. Color images available online at www.liebertonline.com/ast

\begin{align*}
{n_{\rm total}} = \sum \nolimits_M {{C_M}} \sum \nolimits_i {{n_{m
, i}}} \tag{1{\rm d}}
\end{align*}

where the thermal Mach number *M* = *v_b_*/*v_M_* is the ratio of bulk to thermal speed ${v_M} = \sqrt {8k{T_m} / \pi m}$. Here, the “normalized” source rate $\Omega$ is the ratio ${ \sigma _i} / {v_M}$ of the jet source rate to thermal speed (the subscript *M* signifies the possible Mach number dependence of *T* and *v*), and we express (Eq. 1d) the total density as the summation over (1) a thermal Mach number distribution *C_M_* (with Σ_*M*_*C_M_* = 1), and (2) all *i* jet sources. The expression assumes radial expansion of the gas from the surface sources at constant speed, neglecting gravity since the mean molecular speed in the jets significantly exceeds (by at least a factor two) the 240 m/s Enceladus escape speed. The summation (1d) approximates the jets as collisionless and noninteracting at the observation altitudes (Table 1), which is justified since the jet widths of a few kilometers are below the ∼10 km molecular mean free path at the maximum ∼3 × 10^14^ m^−3^ H_2_O plume gas densities observed (Fig. 3) at Cassini's 66–90 km altitude by INMS (although the path is short enough that intermolecular collisions between interacting jets likely introduces a minor correction to the plume structure). Equation 1 can be readily evaluated along the Cassini trajectories (or, for UVIS data, integrated numerically along the occultation line of sight), enabling rapid iteration through plume parameter space, that is, source positions, rate, thermal Mach numbers, and jet pointing directions. By fitting these plume source properties, such modeling can yield information important for understanding and constraining the physics within the fissures and that of the gas expansion at the surface.

Our model considers two possible plume contributors: (1) an upward (normally) directed gas source continuously distributed along the tiger stripes, and (2) multiple jets at discrete tiger stripe locations. For a continuous source, the summation, Equation 1d, is formally an integration along the tiger stripes, which we approximate with many (350 in total) closely (2 km) spaced upward-directed jets along the tiger stripes as done by Tenishev *et al.* (2014). The continuous source, or the jets with locations/pointing from the work of Porco *et al.* (2014), are given a thermal Mach number distribution, as appropriate for gas emerging from a fissure with a distribution of flow velocity (*e.g.*, slower near the walls [Tucker *et al.*, 2015]). We use a four-point Mach number distribution from Mach 0, 2, 4, and 16, respectively; that is, a mixture of thermal isotopically expanding gas (Mach 0) to fast supersonic emission (Mach 16), as required to best fit the shapes of the features in the UVIS and INMS data (Fig. 10). We performed multiple model runs by varying each of the four Mach number contributions (*i.e.*, a four-dimensional parameter space) and determined the relative source fluxes that provided the best fit to all of the (INMS and UVIS) flyby data sets. The best fit values are 18% (at Mach 0), 18% (at Mach 2), 52% (at Mach 4), and 12% (at Mach 16), with ∼±30% uncertainty on each contribution. The Mach numbers 0, 2, 4, and 16 apply to CO_2_; for H_2_O, we assume the bulk gas speed and temperature to be equilibrated with CO_2_ and, accordingly, scale the thermal Mach numbers down in inverse proportion to the (mass dependent) thermal velocity, that is, by (m_H2O_/m_CO2_)^1/2^ = 0.64 (Hurley *et al.*, 2015; Perry *et al.*, 2015). The Mach number distribution is described by the source flux weights *C_M_* (Eq. 1d) for each Mach number, which we set to *C*_1_ = 0.18, *C*_2_ = 0.18, *C*_3_ = 0.52, and *C*_4_ = 0.12, as shown in Figure 10. These values feed into the expression for the total source rate *S* from the Enceladus plume:

\begin{align*}
S = \sum \nolimits_i {{ \Omega _i}} \sum \nolimits_M {{C_M}{v_M}.} \tag{2}
\end{align*}

The model is fit to each INMS or UVIS data set (Table 1) by varying the strength (*i.e.*, ${ \Omega _i}$) of the emission versus position along the tiger stripes (model 1), or the strengths of the jets (model 2), with a regression analysis. While we allow for changes in jet strengths, we approximate the jet directions as reported by Porco *et al.* (2014) to be fixed in time (although we do not rule out directional variability within the reported uncertainty). For INMS data, we only fit sources close enough to the spacecraft to contribute significantly to the density at Cassini's position, such that the uncertainty in the fitted source strength is reasonably small. Distant sources with uncertainty exceeding ±100% are all given the same intensity, and are adjusted together, in unison, in the fitting procedure. The regression analysis yields multiple solutions that correspond to reductions/enhancements in different combinations of jets, or different vapor source distributions along the tiger stripes. These solution families are unique to each INMS/UVIS flyby observation; we did not find any combination of jet strengths that could simultaneously fit multiple flybys. In this article, we average (for each flyby) the model solutions to obtain a consensus estimate of tiger stripe emission profile (model 1) or set of jet intensities (model 2) that fit the flyby data, and the associated margin of error, respectively, versus (1) position along the tiger stripe, or (2) for each jet. For example, if all (or most) model solutions require the enhancement of a specific jet (or jets) to fit the data, this fact will be reflected in the consensus solution.

## 3. Results and Interpretation

**FIG. 4.**
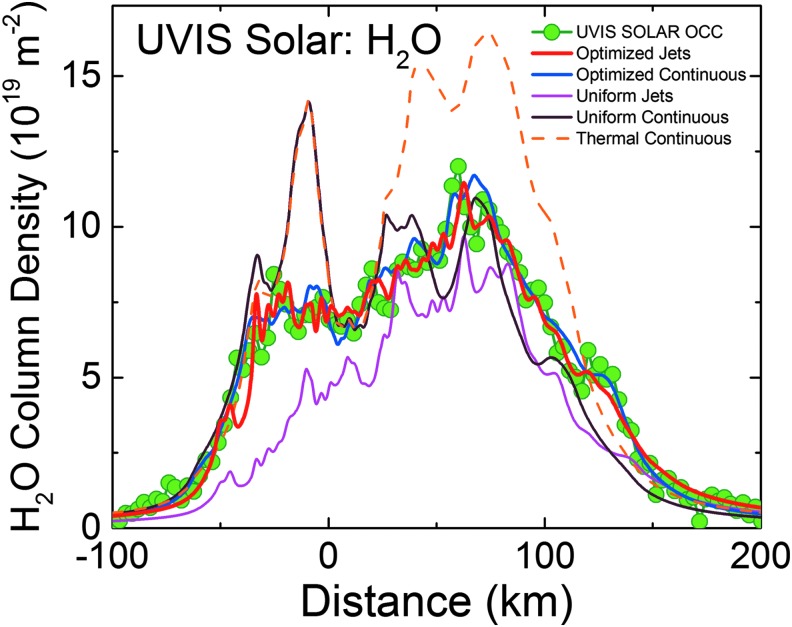
Enceladus plume water vapor column molecular number density measurement from the UVIS 2010 solar occultation (green circles). The *x*-axis origin is the point of closest approach (CA) of the line-of-sight to the sun to the limb of Enceladus (at 14 km altitude); *x*-axis gives the minimum distance of the line-of-sight from CA (time increases left to right, see also Figs. 5–6). We used the Hansen *et al.* (2011) calibration to estimate the H_2_O column density. Red line: average model solution for the optimized (Porco *et al.*, 2014) jets. Blue line: average solution for continuous emission along the tiger stripes. Magenta line: jets with equal intensity. Brown line: uniformly distributed continuous curtain emission along the tiger stripes; this model predicts four large peaks coincident, from left to right, with the Alexandria, Cairo, Baghdad, and Damascus tiger stripes. Orange dashed line: continuous curtain emission with emission strength dependent on tiger stripe temperature (proportional in this example to *T^n^*, with *n* = 7). Color images available online at www.liebertonline.com/ast

**FIG. 5.**
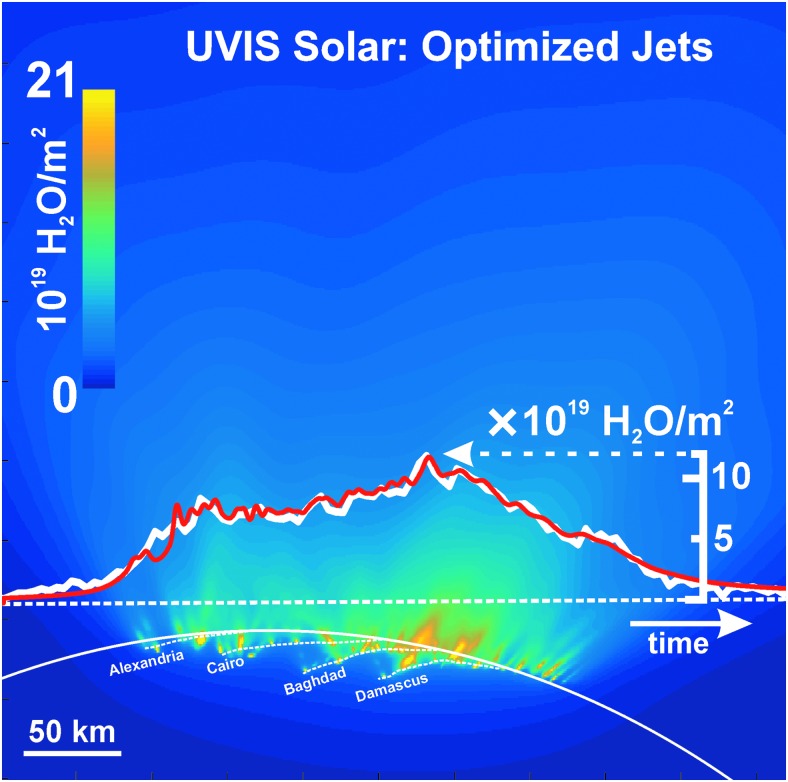
Modeled plume water vapor column density for the optimized (Porco *et al.*, 2014) jets, as viewed along Cassini's line of sight to the sun during the UVIS 2010 solar occultation. Dashed line: line scanned by UVIS, versus time from left to right. White line: H_2_O column density profile measured by UVIS versus position along the scan line (see scale on right). Red line: modeled H_2_O column density profile versus position along the scan lines; note coincidence of peak positions with locations of jets along the scan line. Profiles are same as those in Figure 8. Color images available online at www.liebertonline.com/ast

**FIG. 6.**
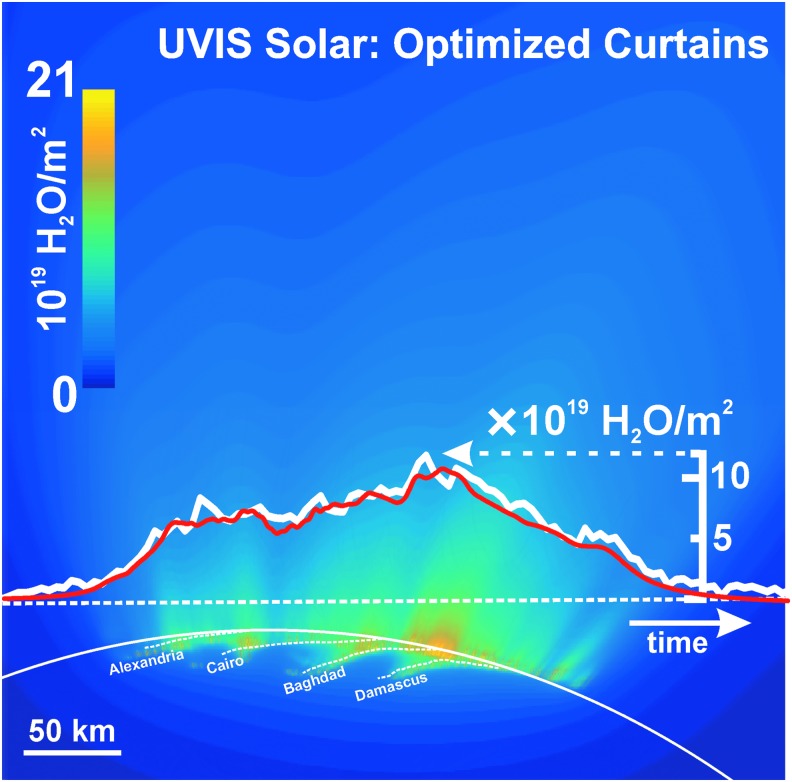
Same as Figure 5 for optimized continuous curtain emission along the tiger stripes, which we approximated by 350 closely (2 km) spaced upward-directed jets along the tiger stripes. Color images available online at www.liebertonline.com/ast

**FIG. 7.**
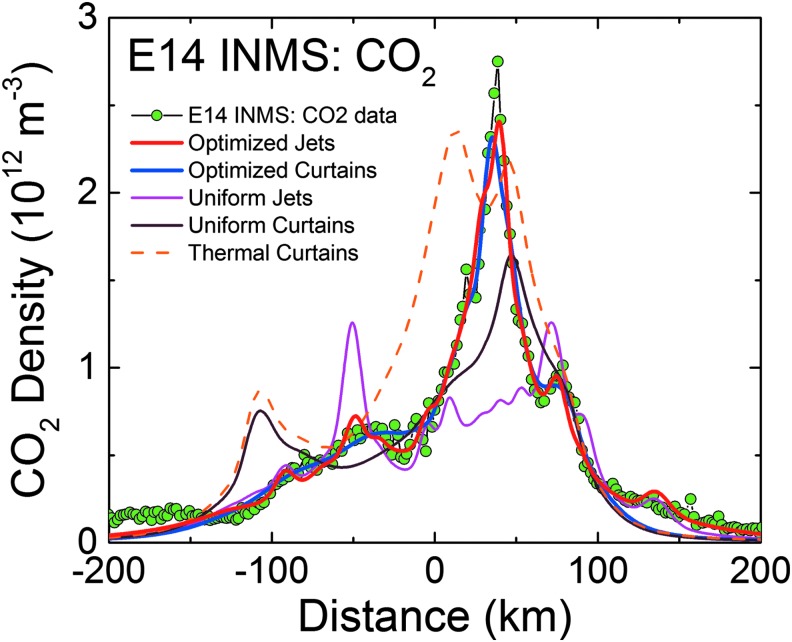
INMS CO_2_ molecular number density measurement (green circles) along the 90 km altitude E14 flyby trajectory showing structure in the along-track plume density. Results plotted versus distance from closest approach to Enceladus along Cassini's trajectory, with time increasing left to right. Red line: average model solution with the (Porco *et al.*, 2014) jets as the constraint. Blue line: average solution for continuous emission along the tiger stripes. Magenta line: jets with equal intensity. Brown line: uniformly distributed continuous curtain emission along the tiger stripes. Orange dashed line: continuous curtain emission with emission strength dependent on tiger stripe temperature (proportional in this example to *T^n^*, with *n* = 7). We used the processed CO_2_ data from the work of Perry *et al.* (2015), and the updated INMS neutral density calibration model from the work of Teolis *et al.* (2015), to estimate the gas density. Note that the average CO_2_ mixing ratio is measured by INMS to be ∼0.24% in the plume (in terms of molecular density), with H_2_O vapor comprising most of the remaining gas (∼90%). Color images available online at www.liebertonline.com/ast

**FIG. 8.**
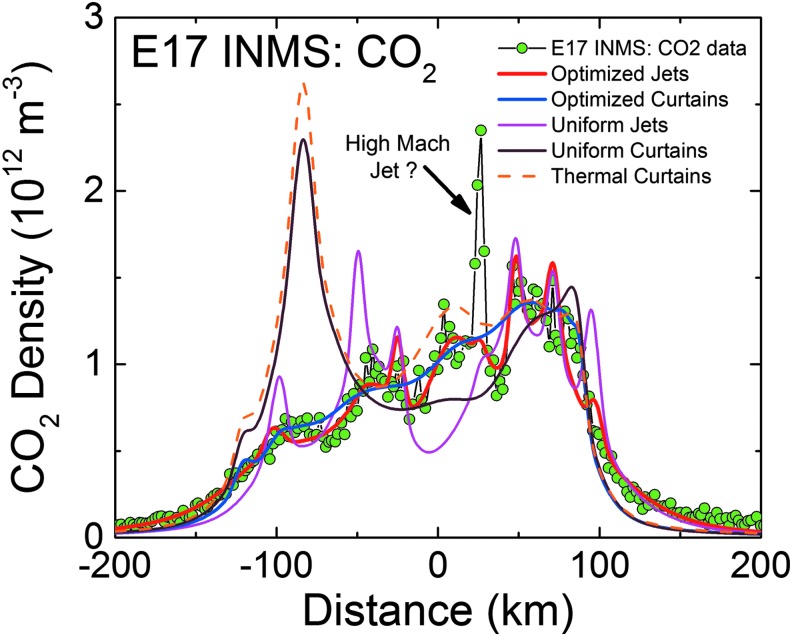
Same as Figure 7 for the 66 km altitude E17 flyby. The CO_2_ mixing ratio in the plume vapor is measured by INMS to be ∼0.37%. The sharp peak at ∼30 km past closest approach, not captured by the models, may be high Mach jet directly intercepted by Cassini, or (less likely) an ice grain impacting the instrument not filtered by the data processing (Perry *et al.*, 2015). Color images available online at www.liebertonline.com/ast

**FIG. 9.**
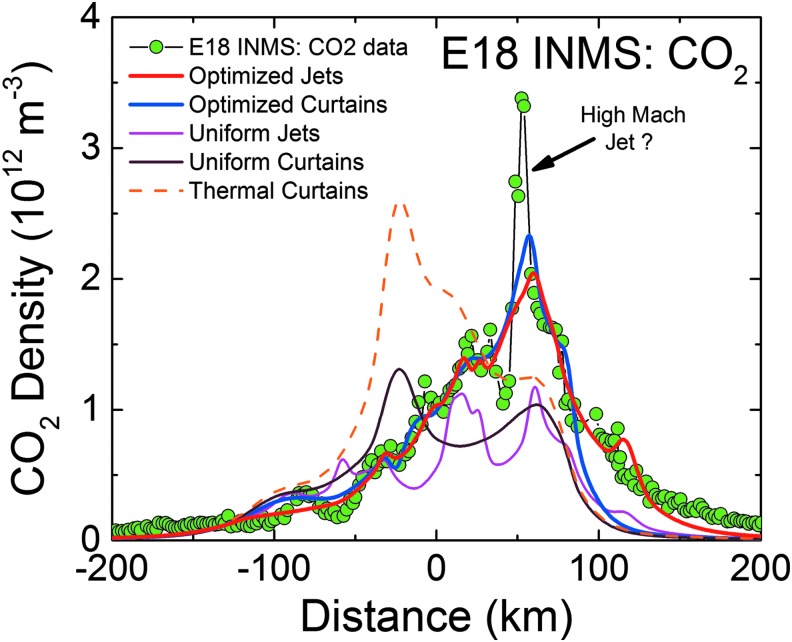
Same as Figures 7and 8 for the 66 km altitude E18 flyby. The CO_2_ mixing ratio in the plume vapor is measured by INMS to be ∼0.24%. The sharp peak at ∼50 km past closest approach, not captured by the models, may be high Mach jet directly intercepted by Cassini, or (less likely) an ice grain impacting the instrument not filtered by the data processing (Perry *et al.*, 2015). Color images available online at www.liebertonline.com/ast

**FIG. 10.**
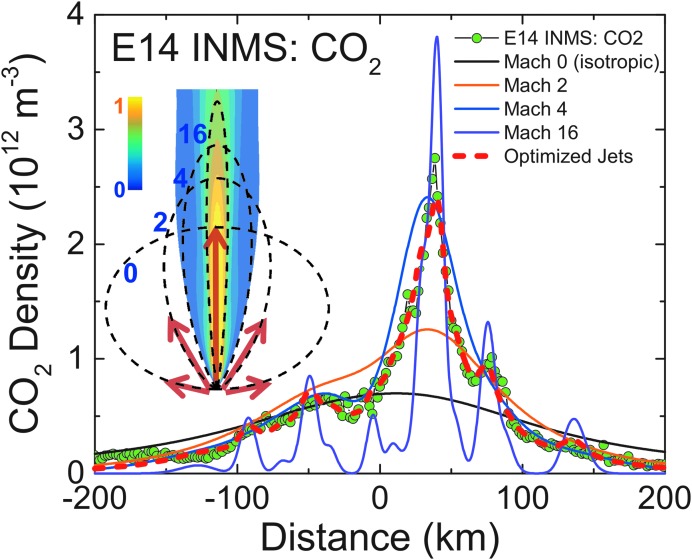
Example (with INMS E14 CO_2_ data: green points) showing why a Mach number distribution in the gas source is needed to fit data. Red dashed line: model fit using the (Porco *et al.*, 2014) jets, assuming a distribution of Mach number in the normalized jet source flux (*Ω_M_* = S/*v_M_*, see text) with 18%, 18%, 52%, and 12% contributions from thermal Mach numbers 0, 2, 4, and 16, respectively. Solid lines: same model, except that all of the gas flux is at the given Mach number; black: 0, orange: 2, blue: 4, violet: 16. To aid in comparing the curves we have rescaled the Mach 0 and 16 curves by factors 3 and 0.5. Inset: schematic representation showing how the emission flux angular distributions (dashed lines) at different Mach numbers are summed to produce the modeled jet (density cross section contours shown, densities and dimensions to scale in relative units). Color images available online at www.liebertonline.com/ast

**FIG. 12.**
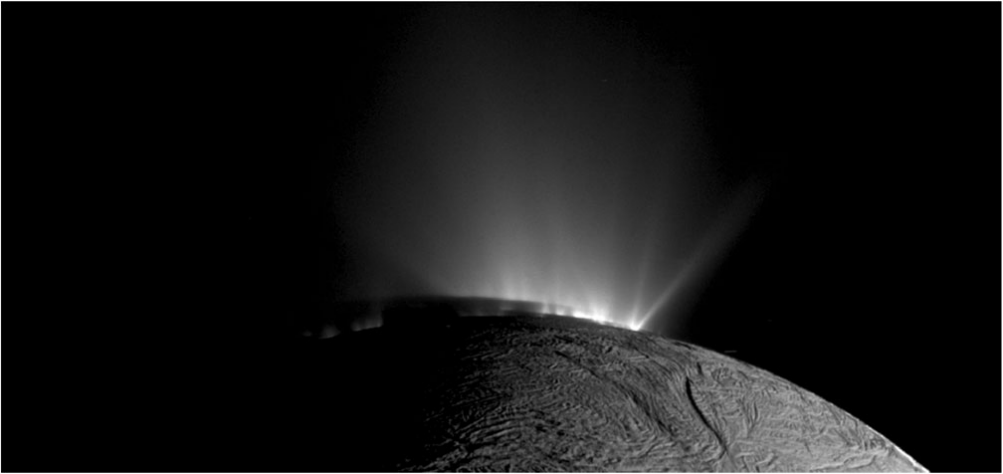
ISS high-resolution (400 m/pixel) image mosaic of the gas-propelled ice grain emission along the tiger stripes, showing the plume source to be characterized by numerous normally and non-normally pointed high Mach jets, together with sheets of continuous interjet emission. Note the near-surface brightness in between the jets just beyond (forward of) the terminator, indicating the presence of gas-propelled ice grains in both discrete and continuous, interjet, fissure-like eruptions. Figure reproduced from the work of Porco *et al.* (2014).

In Figures 3–9, we compare two end-member models—the continuous emission and nominal 98-jet model—to the INMS E14, 17, and 18 CO_2_ density measurements and to the UVIS solar occultation H_2_O column density measurements, and we find that both models succeed in approximating the broad and the fine-scale structures (attributable to localized sources such as individual jets) observed in the gas densities. The gas distributions from the tiger stripes and jets are well blended at Cassini's altitude (*e.g.*, 66 km E17, 18 flybys) due to the angular spread of the emission and, therefore, INMS is to a large degree measuring a summation over multiple tiger stripes and gas jets. We also show in Figure 3 the modeled H_2_O densities along the E14, 17, 18 trajectories, estimated on the basis of the model fits to CO_2_, after correcting for the relative mixing ratios of CO_2_ (0.4% ± 0.1%, depending on the flyby) and H_2_O (∼90%) and the molecular mass dependence (m_H2O_/m_CO2_)^1/2^ of the thermal Mach number (Hurley *et al.*, 2015; Perry *et al.*, 2015). The significant disagreement of the INMS H_2_O measurement with the model is due to H_2_O adsorption in the instrument, which distorts the signal by delaying the transmission of water vapor through the INMS (Teolis *et al.*, 2010). Some features of the data are missed by both the continuous and the jet models, most notably the intense sharp peak in the E17 CO_2_ density (Figs. 3 and 8), and another at E18 (Figs. 3 and 9), possibly (though not likely) due to an ice grain impact in the INMS not filtered by the data analysis (Perry *et al.*, 2015), or (more likely) due to Cassini directly intercepting individual high Mach number jets. These jets (1) may have been missed by our model as a result of the reported uncertainty (Porco *et al.*, 2014) in the jet pointing directions, due to error inherent in the triangulation, together with a possible contribution of real jet directional variability, or (2) these may be unidentified jets missed by ISS imaging due to stochastic variability in their emission strength as discussed by Porco *et al.* (2014). Stochastic variability may result from clogged vents blowing open under the build-up of pressure, downward propagating cracks meeting a water-filled crack leading to a violent explosive outgassing, or pressure-driven fluid motion leading to boiling in tidally flexed cracks as discussed below. However, the nominal jet model captures some aspects that continuous emission does not, particularly the outbound CO_2_ tails in the E14, 17, and 18 INMS data (Figs. 3 and 7–9). The tails are successfully fit with several of the high-angle (off-normally pointed) (Porco *et al.*, 2014) jets, suggesting that a fraction of the plume gas is ejected (Porco *et al.*, 2014) with significant off-normal bulk velocity components. A model consisting of only jets is sufficient to explain the data, provided the jet sources contain (in addition to the sharp high Mach number emissions) a component of slow, isotropic gas emission, as shown in Figure 10. However, the most plausible interpretation of the UVIS and INMS data is that the plume vapor distribution is at least partially attributable to jets, possibly mixed with continuous interjet emission along the tiger stripes. We reproduce Figure 12 from the work of Porco *et al.* (2014) showing the highest resolution (80 m/pixel) ISS image mosaic, in which it can be seen that a combination of both (1) numerous off-normal jets and (2) continuous sheets of interjet brightness due to faint particulate sheets most accurately describe the plume source.

The mixture of both high and low Mach number components in the gas flux escaping the surface fissures, as implied by the UVIS and INMS data (Fig. 10), suggests that the emission may contain two contributions as follows: (1) fast vapor preaccelerated by the pressure gradient along the fissure length (or through nozzle-like throats within the fissure [Schmidt *et al.*, 2008; Yeoh *et al.*, 2015]) and (2) slow thermalized gas, which has either not undergone acceleration in the fissures (*e.g.*, a near-surface liquid or solid [Goguen *et al.*, 2013] sublimation source) or has been rethermalized by friction and/or thermal exchange with the fissure walls. The thermalized component is still at collisional densities of order 10^21^ molecules/m^3^ on emergence from the surface vent (Tucker *et al.*, 2015) and thereby undergoes collisional expansion up to a few kilometers from the source (Yeoh *et al.*, 2015). The millimeter gas mean free paths at the surface vent are shorter than the fissure widths, which are possibly tens of centimeters (Schmidt *et al.*, 2008; Yeoh *et al.*, 2015; Kite and Rubin, 2016), and thus the flow may segregate in the channel, with fast (cold) and slow (thermalized) vapor concentrated in the channel center and at a boundary layer near the fissure walls, respectively (Tucker *et al.*, 2015). Gases such as CO_2_ measured by INMS are entrained in the water vapor flow, both near the walls and channel center, and thereby emerge from the fissures with bulk flow speed approximately equilibrated to that of the water vapor.

A limitation of the INMS (UVIS) gas density (column density) data is that it only provides constraints on the gas thermal Mach number, that is, the ratio of the bulk-to-thermal speed, whereas the gas bulk speed and temperature entering into the ratio are not constrained. The uncertainty is particularly acute in the high Mach number (fast) component of the emission, since it is not clear whether the gas is accelerated purely by passive adiabatic expansion of vapor from a static source (clathrates, or a stationary liquid reservoir at the H_2_O triple point; 611 Pa and 273 K), or whether the fluid acceleration is augmented by tidally driven compressional stress on the fissures (Kite and Rubin, 2016). Such stress would exert pressure on the fluid, which may flow in response both down into the subsurface ocean (if the fissure connects to the ocean) and transiently up toward the surface (if the fissure connects to the surface). A finite thickness at the top of the rising liquid column may boil as the pressure acting on the liquid falls below its vapor pressure (Porco *et al.*, 2006; Ingersoll and Nakajima, 2016), drawing latent heat from the liquid to form ice near the triple point (possibly a gas-propelled mist of frozen droplets or grains). The gas/liquid/solid mixture may volumetrically expand nonadiabatically, with the expanding water vapor bubbles drawing heat from the liquid, while exerting (together with exsolving bubbles of other gas species: CO_2_, H_2_, NH_3_, or CH_4_ [Matson *et al.*, 2012; Bouquet *et al.*, 2015]) additional pressure to accelerate the fluid to high speed before arrival at the surface. For the baseline case of a static subsurface reservoir at the triple point, the gas is accelerated purely by the conversion of gas thermal energy to dynamic pressure as discussed by Yeoh *et al.* (2015) and is thus limited to a maximum ∼1 km/s ultimate adiabatic expansion speed at the fissure exit (Ingersoll and Pankine, 2010). However, if tidally driven flow and boiling also contribute to the acceleration, the speed may be much higher; for example, boiling liquid rising close to the surface may yield vapor escaping to space near 273 K, which (for Mach 16 × (18/44)^1/2^ ≈ 10 flow as seen by UVIS/INMS) implies a flow speed of ∼6 km/s. This translates to a ∼3 cm/s liquid flow speed in the fissures after dividing by the ∼2 × 10^5^ liquid-to-gas volume expansion factor; this is consistent with the required speed, ≥1.3 cm/s, for water to transit a 10 km thick ice shell (Thomas *et al.*, 2016) within the freezing time scale (∼10^6^ s for *w ∼*1 m, scales as fissure width squared [Porco *et al.*, 2014]). This speed is a lower limit since some liquid freezes instead of vaporizing, and the remaining liquid may not have sufficient time to fully vaporize before exiting the vent. Hence, higher liquid flow speeds, for example, a few tens of centimeter per second as estimated by Kite and Rubin (2016) for 1 m fissures, could plausibly generate high-speed (∼6 km/s) jets with the thermal Mach numbers seen by UVIS and INMS. However, we note that the plume scale height appears constant in VIMS (with the possible exception of a small range of orbital position around apoapse) (Hedman *et al.*, 2009) and ISS (Nimmo *et al.*, 2014) images, not following the plume strength dependence on mean anomaly, which may be evidence that supports the adiabatic expansion scenario, that is, nozzle choked gas flow limited to the ∼1 km/s ultimate expansion speed, independent of source strength.

Accordingly, we have two limiting cases for the relationship of the gas thermal speed *v_M_* exiting the vent to the thermal Mach number: (1) complete adiabatic expansion

\begin{align*}
 { v_M } = { v_0 } \sqrt { \frac { \beta }  { { { M^2 } + \beta } } , } \tag {3{\rm a}}
\end{align*}

obtained (see Gombosi [1994] Eq. 7.57, also Sutton and Biblarz [2001] Eqs. 3–12) from the definition *M* = *v_b_*/*v_M_* and by equating the total energy per molecule *mv_b_*^2^/2 + *mβv_M_*^2^/2 exiting the vent to the stagnation energy *mβv_0_*^2^/2 of the subsurface vapor (here *v_0_* = 565 m/s is the mean thermal speed of H_2_O at 273 K, and *β* = γ/(γ−1) = 4, with γ = 4/3 the water vapor heat capacity ratio); and (2) pure nonadiabatic acceleration (no expansional cooling):

\begin{align*}
{v_M} = {v_0}. \tag{3{\rm b}}
\end{align*}

Substituting into Equation 2 (and using the definition Σ_*M*_ C_*M*_ = 1), we obtain the adiabatic and nonadiabatic plume source rate estimates *S*_a_ and *S*_na_:

\begin{align*}
{S_{\rm a} = B{v_0} \sum \nolimits_i {{ \Omega _i} = B{S_{\rm
na}}} \tag{4{\rm a}}}
\end{align*}

\begin{align*}
{S_{\rm na}} = {v_0} \sum \nolimits_i {{ \Omega _i} , } \tag{4{\rm
b}}
\end{align*}

where, using *C_M_* = [0.18, 0.18, 0.52, 0.12] and *M* = [0, 2, 4, 16] × (m_H2O_/m_CO2_)^1/2^ for water molecules, we find *S*_a_ to be only ∼67% of *S*_na_:

\begin{align*}
B = \sum \nolimits_M { { C_M } } \sqrt { \frac { \beta }  { { {
M^2 } + \beta } } } = 0.67. \tag {4{\rm c }}
\end{align*}

**FIG. 11.**
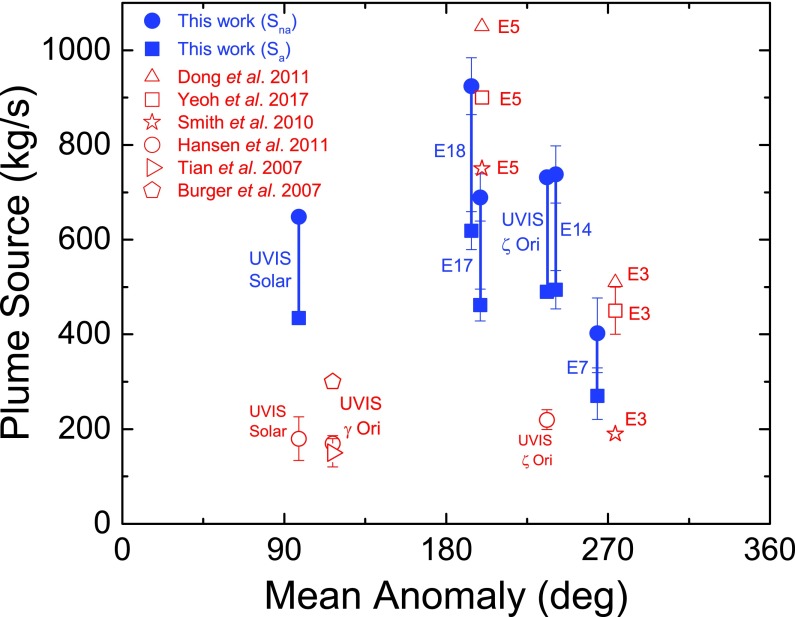
Total plume vapor mass source rates for the UVIS zeta Orionis and solar occultations, and the E14, E17, and E18 flybys, from our modeling (blue symbols), and for E3 and E5 from the works of Dong *et al.* (2011), Smith *et al.* (2010), and Yeoh *et al.* (2017), versus Enceladus mean anomaly at the time of the measurement. For our results, we show both *S_na_* (solid circles) and *S_a_* (solid squares) nonadiabatic and adiabatic upper and lower limiting source rates, and vertical lines to show the range of possible source rates. For the UVIS zeta Orionis and solar data, Hansen *et al.* (2011) used a 450 m/s gas speed (the H_2_O 170 K thermal speed) to estimate 220 and 180 kg/s source rates; our estimates are at least a factor two higher since our model requires significant high Mach gas flow. The source rate uncertainty and stochasticity, and the scarcity of measurements near periapsis, prevent us from conclusively identifying a mean anomaly trend. Color images available online at www.liebertonline.com/ast

The plume source rate estimate therefore depends on the assumptions regarding the gas temperature and speed in the high Mach gas component of the plume. This is shown in Figure 11, where we plot the INMS- and UVIS-based plume source rate estimates versus Enceladus mean anomaly, for both the *S* = *S*_a_ and *S* = *S*_na_ limiting cases.

We also contrast in Figures 4 and 7–9 the continuous emission and jet models, optimized to fit the UVIS and INMS data, with three other (nonoptimized) models: (1) the Porco *et al.* (2014) jets with equal intensity, (2) continuous emission with uniform intensity along the tiger stripes, and (3) continuous emission correlated to the average tiger stripe temperature as estimated by the CIRS team (Spencer *et al.*, 2013) from the radiated thermal brightness detected by CIRS versus position along the tiger stripes (Howett *et al.*, 2011). As discussed by Spencer *et al.* (2013), the temperatures and widths of the endogenic tiger stripe emission (modeled with a wide low-temperature *T_l_*, and a narrow high-temperature component *T_H_*, as continuous “bands” along the tiger stripes [Spencer *et al.*, 2013]) are optimized to best match the stripe's infrared thermal emission spectra. The most extensive spatial coverage (obtained in March 2008 [Howett *et al.*, 2011]) of the tiger stripes by CIRS FP3 (600–1100 cm^−1^) and FP4 (1100–1400 cm^−1^) is used. The FP3/4 sensitivity only allows for constraints on the high-temperature endogenic component and, therefore, the low-temperature component is constrained by a CIRS FP1 (10–600 cm^−1^) scan across Damascus, Baghdad, and Cairo obtained in August 2010 (Spencer *et al.*, 2013) (their Fig. 1). Since the CIRS spatial resolution does not permit identification of discrete hot spots in most of the tiger stripe system, this model is best suited to modeling a background of continuous or diffuse gas emission along the tiger stripes, rather than individual jets. Our approach was to define an empirical relationship positively correlating gas source rate to the temperature *T_H_* (*i.e.*, the high-temperature component, presumably concentrated at the vents), then to adjust parameters to determine whether a fit to the data was possible (as an example we show a power law relationship in Figures 4, and 7–9).

We found (Figs. 4 and 7–9) that no choice of functional relationship of source rate to temperature provided a good fit to the observations. Although warm fluid flow (gas and/or liquid) through the fissures is a plausible explanation for surface heating around the surface vents (Matson *et al.*, 2012) (whether by condensation latent heat or direct conduction to the walls), the thermal inertia of the surface material (≥27 Jm^−2^Ks^−1/2^ [Howett *et al.*, 2011]) may make the surface temperatures around the vents unresponsive to rapid change (on the order of hours or less) in local emission strength. Therefore, irrespective of the correlation of tiger stripe temperatures to the time-averaged plume activity (Porco *et al.*, 2014), the tiger stripe temperatures may not well approximate the local emission strength at any given moment. Additionally, the relationship of temperature and gas flux may be a function of geometrical considerations at the vent. For a tidally flexed planar fracture, it is possible the fluid flux could vary proportionally to the fracture width, a result consistent with choked gas flow, leaving the contact area between the gas and the fracture wall (and thereby the heat flux) independent of width.

**FIG. 13.**
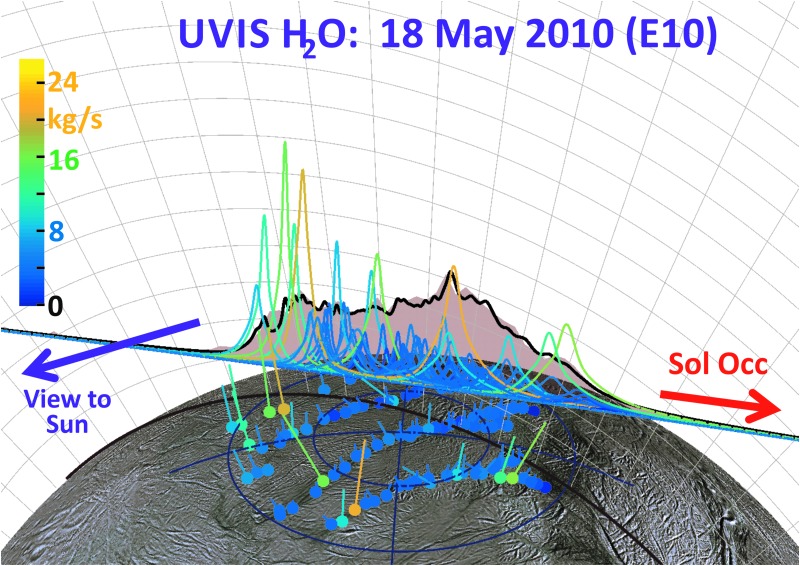
To scale 3D representation of the 2010 Enceladus plume UVIS Solar Occultation with vertical areas representing (in linear scale) the occulted intensity fraction (corresponding to the water vapor column density), and the flat base of the area corresponding to the line of minimum ray height (brown line on surface is the “ground track” of this ray). Cassini's viewpoint onto the plume is from the upper right of the figure, and UVIS is scanned from left to right as shown by the arrow. Dots on surface: (Porco *et al.*, 2014) jet source locations, with straight lines showing jet directions. The colors and jet line lengths are given by the optimized jet strength: orange (blue), long (short) jets represent high (low)-intensity jets. Color bar scale: estimated jet source rates in kilograms per second. Colored curves: line height gives the estimated column density profile of each jet along the UVIS line of sight. Black curve: the best fit total column density after summing the contributions of all jets. Color images available online at www.liebertonline.com/ast

**FIG. 14.**
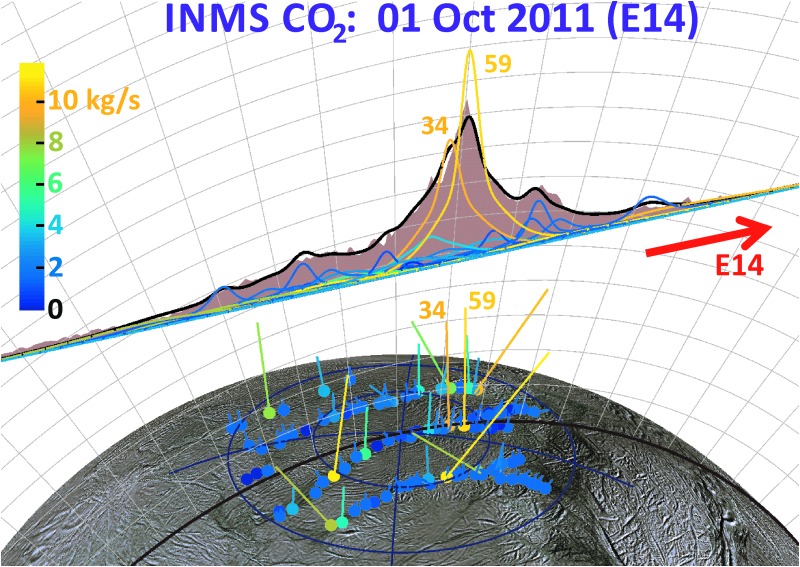
To scale 3D representation of the INMS CO_2_ data from the 90 km E14 flyby, with vertical areas representing (in linear scale) the mass 44 CO_2_ signal (corresponding to the CO_2_ gas density), and the flat base of the area corresponding to Cassini's trajectory (red arrow shows spacecraft direction, brown line on surface is the ground track). Dots on surface: (Porco *et al.*, 2014) jet source locations, with straight lines showing jet directions. The colors and jet line lengths are given by the optimized jet strength: orange (blue), long (short) jets represent high (low)-intensity jets. Color bar scale: estimated jet source rates in kilograms per second, renormalized to correct for the measured 0.24% CO_2_ mixing ratio in the plume. Colored curves: line height gives the estimated density profile of each jet along the E14 trajectory. Black curve: the best fit total column density after summing the contributions of all jets. The model suggests enhanced emission from jets 34 and 59 (Porco *et al.*, 2014) elevated activity, as these are the only jets with the required location and pointing to fit the intense CO_2_ density maximum. Color images available online at www.liebertonline.com/ast

**FIG. 15.**
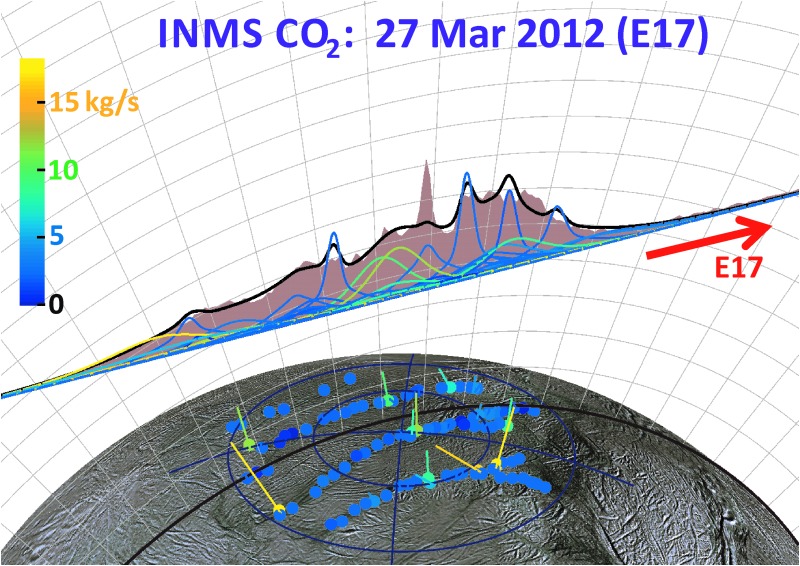
Same as Figure 14 for the 66 km altitude E17 flyby. Color images available online at www.liebertonline.com/ast

**FIG. 16.**
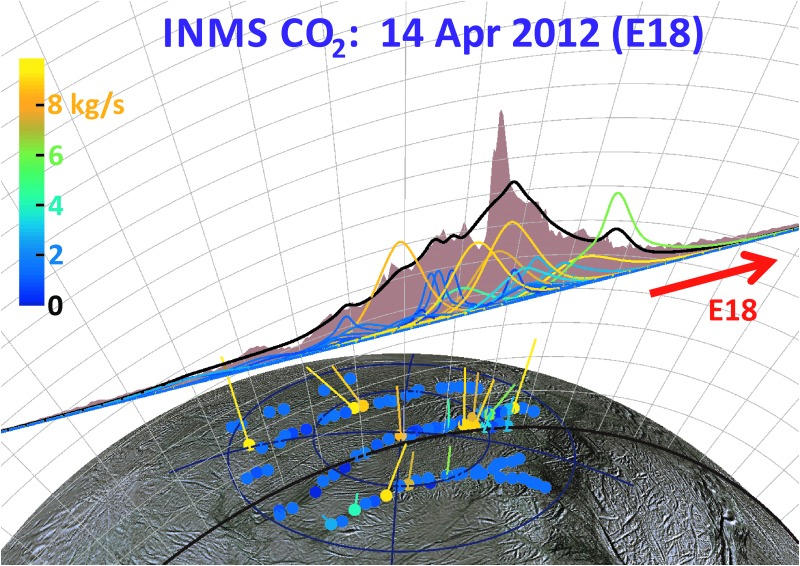
Same as Figures 14 and 15 for the 66 km altitude E18 flyby. Color images available online at www.liebertonline.com/ast

As can be seen by comparing the 3D projections of Figures 13–16, our model results suggest drastic time variability in the plume sources along the tiger stripes. Using the Porco *et al.* (2014) jets as the modeling constraint, the model allows, in some cases, identification of specific jets that may have changed between flybys. An example is jets 37 and 59 from the work of Porco *et al.* (2014); only these jets have the required location and direction to fit the major peak in the E14 CO_2_ density, suggesting enhanced activity from these jets during E14 (Fig. 14), with source rates ∼10 and ∼12 kg/s, respectively (significantly above the average ∼1.5 kg/s intensity of the other jets). At these intensities, however, jets 37 and 59 would contribute too much signal to the E17 model, that is, the model fit to the E17 data requires these jets to be at low intensity during the flyby (Fig. 15). Many vapor jets appear to change drastically and stochastically between flybys, showing no discernible correlation to the nominal jet sightings in ISS images, or their proxy intensities as reported by Porco *et al.* (2014). This could be due to inherent and even expected changes, either in the strength or the direction of the jets, between the times the various instruments made their observations. As can be seen in Figure 11, the total plume source rate also exhibits substantial stochastic variability, up to a factor ∼5 between measurements, and most of the measurements are clustered in a small range of mean anomaly just past 180° apoapsis, making any systematic mean anomaly dependence in the vapor emission difficult to discern.

## 4. Conclusions

The Cassini spacecraft's multiple low-altitude traversals through the Enceladus plume, and the direct capture and high-cadence analysis of gas intercepted by INMS along the spacecraft trajectory, have revealed in high resolution the structurally complex and dynamic vapor cloud emanating from discrete plume sources (tiger stripes, geysers). The extraction from INMS of plume densities, both for minor species and for sticky water vapor, has proved to be a major challenge, spanning many years and multiple flyby attempts and requiring the development of new instrument calibration models (Teolis *et al.*, 2015) and data analysis methods (Teolis *et al.*, 2010; Perry *et al.*, 2015). On the basis of the INMS data, in combination with UVIS stellar and solar occultation measurements of the vapor column density through the entire thickness of the plume, our models reveal that the plume's vapor distribution requires both low and high Mach emission from the surface sources, a result consistent with a mix of (1) high-speed gas emission (*e.g.*, thermal expansion of gas through nozzle-like channels or boiling/pressure-driven acceleration in narrow fissures) and (2) low-speed thermal emission, such as a near surface solid or liquid sublimation source or a thermalized gas layer in contact with the fissure walls. The INMS and UVIS data suggest the presence of multiple discrete and off-normal gas jets, likely combined with continuous interjet emission, a finding consistent with high-resolution images (Porco *et al.*, 2014) (Fig. 12). Combined with observations acquired along closely spaced trajectories but at different times, the modeling also implies drastic and stochastic time variability of the individual gas jets and/or distribution of emission along the tiger stripes, consistent with ISS indications of stochastically time-variable jets (Porco *et al.*, 2014). The total plume intensity, between 100 and 1000 kg/s, is also stochastically variable between Cassini flybys in UVIS and INMS observations, and estimates of the source rate are dependent (by up to ∼30%) on the assumed temperature and speed of the high Mach emission, as determined by the roles of adiabatic and nonadiabatic fluid expansion in accelerating the gas. The confirmation by multiple Cassini instruments of such intricate structure and dynamics in the Enceladus plume is a seminal accomplishment of Cassini's decade-long exploration of this active world, and a compelling basis upon which to elucidate, by way of earth-based studies and future spacecraft exploration, the origin and physics of the Enceladus plume and its physical and compositional relationship to the subsurface ocean.

## Abbreviations Used

3D: three-dimensional
INMS: ion neutral mass spectrometer
UVIS: ultraviolet imaging spectrograph
